# Exploring the link between perceived physical literacy and academic performance outcomes: insights from the EHDLA study

**DOI:** 10.3389/fspor.2024.1352114

**Published:** 2024-01-24

**Authors:** Gabriel Domínguez-Martín, Pedro J. Tárraga-López, José Francisco López-Gil

**Affiliations:** ^1^ Consejería de Educación, Region of Murcia, Murcia, Spain; ^2^ Departamento de Ciencias Médicas, Facultad de Medicina, Universidad de Castilla-La Mancha, Albacete, Spain; ^3^ One Health Research Group, Universidad de Las Américas, Quito, Ecuador

**Keywords:** grade point average, academic achievement, school performance, language, maths, foreign language, physical activity, physical education

## Abstract

**Objective:**

The aim of this study was to verify the relationship between perceived physical literacy (PPL) and academic performance outcomes among Spanish adolescents aged 12–17 years.

**Methods:**

This cross-sectional study is a secondary examination utilizing data derived from the Eating Healthy and Daily Life Activities (EHDLA) project. The Spanish Perceived Physical Literacy Instrument for Adolescents (S-PPLI) was used to evaluate the PPL of the participants. To determine academic performance, the numerical grades for each subject were added together and then divided by the total number of subjects.

**Results:**

Overall, a positive trend in the association between S-PPLI scores and academic performance was observed. We identified two significant ranges within the S-PPLI scores. First, there was a significant range between 9 and 33 points on the S-PPLI, indicating an inverse association with academic performance. Second, another significant range was observed at scores of 34 points or higher on the S-PPLI, suggesting a positive association with academic performance. Participants with low PPL had the lowest academic performance (mean = 6.4; bias-corrected and accelerated (BCa) bootstrapped 95% confidence interval (CI): 6.2–6.6). Conversely, those adolescents with high PPL had the highest academic performance (mean = 6.9; *BCa* bootstrapped 95% CI: 6.6–7.1). Significant differences were found between low PPL and medium PPL (*p*-adjusted = 0.031) and between low PPL and high PPL (*p*-adjusted = 0.031).

**Conclusions:**

Increased physical literacy could be a relevant factor for achieving greater academic performance in adolescents. This study has potential implications for physical education instructors, school leaders, and healthcare practitioners.

## 1 Introduction

Academic performance in childhood and adolescence has a significant influence on shaping the future path of a young person (1). It forms the basis for individuals' cognitive growth, ability to think critically, and overall academic abilities (2). Academic success goes beyond a student's current educational achievements; it also has a profound impact on their ability to reach long-term goals, succeed in their careers, and achieve overall well-being (3). A strong academic history during these crucial years can enhance self-assurance, expand career prospects, and cultivate a lifelong passion for learning (4). Hence, providing care and encouragement to children's academic path during their early years can result in extensive beneficial effects on their personal, professional, and intellectual growth (5).

There is a growing body of evidence suggesting that encouraging and maintaining healthy lifestyle habits can be a successful strategy for enhancing the academic performance of young individuals (6–8). Recently, there has been a notable increase in the focus on physical literacy, resulting in the creation of numerous initiatives, educational plans, and policies designed to improve it (9, 10). Given these circumstances, it is especially crucial to elucidate the potential outcomes of advocating for physical literacy (11). Physical literacy includes the motivation, self-assurance, physical abilities, knowledge, and comprehension that individuals acquire to sustain a suitable level of physical activity throughout their entire life (12, 13). Physical literacy is cultivated through engagement in physical activities, representing a unique form of intelligence associated with the capacity to execute various motions (14), which goes beyond mere physical effort and serves as a crucial basis for active involvement (15). In relation to its assessment, a review has indicated that most procedures include assessment of the affective and physical domains, while the cognitive domain is less frequently included (16).

To date, only a few studies have evaluated the specific association between physical literacy and academic performance outcomes (17–19). However, considering the strong relationship of physical education (20) or physical activity (21) with physical literacy, it is conceivable that improving individuals' physical activity or physical education (and, by extension, their physical literacy) might lead to greater academic performance (22, 23). In this sense, a systematic review and meta-analysis revealed that quality-focused physical education had a positive impact on academic performance, particularly in mathematics-related skills (22). Furthermore, increasing the amount of moderate-to-vigorous physical activity appears to be associated with beneficial outcomes in terms of core executive function and academic performance (23). Given this lack of studies, gaining deeper insight into this relationship could be valuable in shaping future intervention efforts aimed at improving academic performance in adolescents. Therefore, the aim of this study was to verify the relationship between perceived physical literacy (PPL) and academic performance outcomes among Spanish adolescents aged 12–17 years. We hypothesize that a higher level of physical literacy among Spanish adolescents will be associated with greater outcomes in academic performance based on existing evidence suggesting a relationship between physical activity, physical education, and academic performance.

## 2 Methods

### 2.1 Study design and population

This current cross-sectional study is a secondary examination utilizing data derived from the Eating Healthy and Daily Life Activities (EHDLA) project. The protocol for the EHDLA study has been described previously (24). This study included a representative sample of adolescents aged 12–17 years who were students in three secondary schools located in the *Valle de Ricote* (Region of Murcia, Spain). The data were collected during the 2021–2022 academic year.

The parents or legal guardians of the adolescent participants provided written consent for their involvement in the study. The participants were given comprehensive information about the study's objectives, as well as the assessments and questionnaires that would be carried out. Additionally, the adolescents were asked for their consent to participate. Approval for this study was granted by the Bioethics Committee at the University of Murcia (approval ID: 2218/2018), the Ethics Committee of the Albacete University Hospital Complex, and the Albacete Integrated Care Management (Approval ID: 2021-85). The research adhered to the ethical principles set forth in the Helsinki Declaration.

### 2.2 Procedures

#### 2.2.1 Perceived physical literacy (independent variable)

In this study, the Spanish Perceived Physical Literacy Instrument (S-PPLI) for Adolescents (25) was employed to evaluate the PPL of the participants. The S-PPLI has previously undergone validation for use with Spanish youth. The original Perceived Physical Literacy Instrument (PPLI) was initially designed for physical education teachers and comprises 18 items (26). However, the version adapted for adolescents in this study consisted of nine items. Participants were required to rate these items on a 5-point Likert scale ranging from 1 (strongly disagree) to 5 (strongly agree). The 9 items of the S-PPLI were equally distributed among three categories: knowledge and comprehension, self-expression and interaction with others, and self-perception and self-confidence.

#### 2.2.2 Academic performance indicators (dependent variables)

The assessment of academic performance involved the calculation of the cumulative grade point average (GPA) for all the subjects whom the adolescents had taken. To determine the GPA, the numerical grades for each subject were added together and then divided by the total number of subjects. This calculation resulted in a final average score ranging from 0 (the lowest score) to 10 (the highest score).

#### 2.2.3 Covariates

Information about sex and age was self-reported by the adolescents. To assess socioeconomic status, the Family Affluence Scale-III (27) was used; this scale includes responses from six items related to family possession and amenities, including bedrooms, vehicles, bathrooms, computers, travels, and dishwashers. The Family Affluence Scale-III score ranges from 0 to 13 points, with higher scores indicating a higher socioeconomic status. Adherence to the Mediterranean Diet was assessed using the Mediterranean Diet Quality Index for Children and Adolescents (28). Energy consumption was estimated through a self-administered food frequency questionnaire, which has previously been validated for use in the Spanish population (29). Physical activity and sedentary behavior were assessed using the Youth Activity Profile Physical Questionnaire (30). This self-administered questionnaire covered a 7-day period and included 15 different items categorized into sections such as out-of-school activities, school-related activities, and sedentary habits. To calculate the overall sleep duration, adolescents were asked about their usual bedtime and wake-up times on both weekdays and weekends. The average sleep duration during the week and on weekends was computed using the formula [(average sleep duration on weekdays × 5) + (average sleep duration on weekends × 2)] divided by 7. Body mass index was determined by dividing the participants' body weight in kilograms by the square of their height in meters.

### 2.3 Statistical analysis

For categorical variables, the descriptive statistics included both the count (*n*) and the percentage (%) of observations within each category. For continuous variables, the descriptive statistics included the median and the interquartile range (IQR). To assess the normality of the variables, visual methods such as density and quantile‒quantile plots, as well as the Shapiro‒Wilk test, were used. The associations between the study variables and PPL status (divided into tertiles) were examined using the Kruskal‒Wallis *H* test for continuous variables and the chi-square test for categorical variables. Since preliminary analyses revealed an interaction effect between sex and GPA (*p* = 0.030), we also conducted analyses stratified by sex. To test the relationship between the S-PPLI score and academic performance in adolescents without making any assumptions about the nature of the relationship, generalized additive models (GAMs) were employed. GAMs are versatile models capable of capturing nonlinear relationships in the data without requiring a predefined mathematical structure. In this analysis, the restricted maximum likelihood method was used for selecting the smoothness (31), and a shrinkage approach was applied using thin plate regression spline smoothers (32). The effective degrees of freedom (*edf*) of the GAM were used to quantify the degree of nonlinearity in the relationship. With regard to PPL status (i.e., low PPL, medium PPL, high PPL), an analysis of covariance was conducted to assess its association with academic performance while adjusting for several covariates. A nonparametric bias-corrected and accelerated (*BCa*) bootstrap method with 1,000 samples was employed for this analysis. Following this analysis, we applied a correction for multiple comparisons using the false discovery rate *p* value method, which was developed by Benjamini and Hochberg (33). In addition, we calculated the estimated marginal means of academic performance by S-PPLI score and PPL status. The models were adjusted for several covariates, including sex, age, socioeconomic status, adherence to the Mediterranean diet, energy intake, physical activity, sedentary behavior, overall sleep duration, and body mass index. All the statistical analyses were carried out using R statistical software (version 4.3.2) from the R Core Team in Vienna, Austria, and RStudio (2,023.09.1 + 494) from Posit in Boston, MA, USA. The threshold for statistical significance was set at a *p* value of less than 0.05.

## 3 Results

Table 1 shows the main characteristics of the adolescents analyzed according to PPL status. The greatest GPA was found among adolescents with high PPL (median = 7.2; IQR = 2.7). Conversely, the lowest GPA was observed in adolescents with low PPL (median = 6.4; IQR = 2.2).

**Table 1:** Descriptive data of the sample of adolescents analyzed (*N* = 785).

Variable	Low PPL (9–31 points)	Medium PPL (32–36 points)	High PPL (37–45 points)
Participants (%)	266 (33.9)	285 (36.3)	234 (29.8)
Sex			
Boys (%)	93 (35.0)	139 (48.8)	118 (50.4)
Girls (%)	173 (65.0)	146 (51.2)	116 (49.6)
Age (years)	14.0 (2.0)	14.0 (2.0)	14.0 (2.0)
FAS-III (score)	8.0 (3.0)	8.0 (3.0)	9.0 (3.0)
KIDMED (score)	6,0 (4.0)	7.0 (3.0)	8.0 (3.0)
Energy intake (kcal)	2,644.7 (1,453.2)	2,571.5 (1,475.7)	2,565.4 (1,464.0)
YAP-S physical activity (score)	2.4 (0.9)	2.6 (0.8)	2.9 (0.9)
YAP-S sedentary behaviors (score)	2.6 (0.8)	2.6 (0.8)	2.4 (0.8)
Overall sleep duration (min)	492.9 (77.1)	501.4 (72.9)	501.4 (64.3)
BMI (kg/m^2^)	22.4 (7,0)	21.7 (5.9)	21.0 (5.6)
GPA (score)	6.4 (2.2)	7.0 (2.6)	7.2 (2.7)

The data are reported as the median (interquartile range) or count (percentage). BMI, body mass index; FAS-III, Family Affluence Scale-III; GPA, grade point average; KIDMED, Mediterranean Diet Quality Index in Children and Adolescents; Spanish Perceived Physical Literacy Instrument; YAP-S, Spanish Youth Active Profile. The S-PPLI ranges from 9 to 45 points.

**Figure 1:**
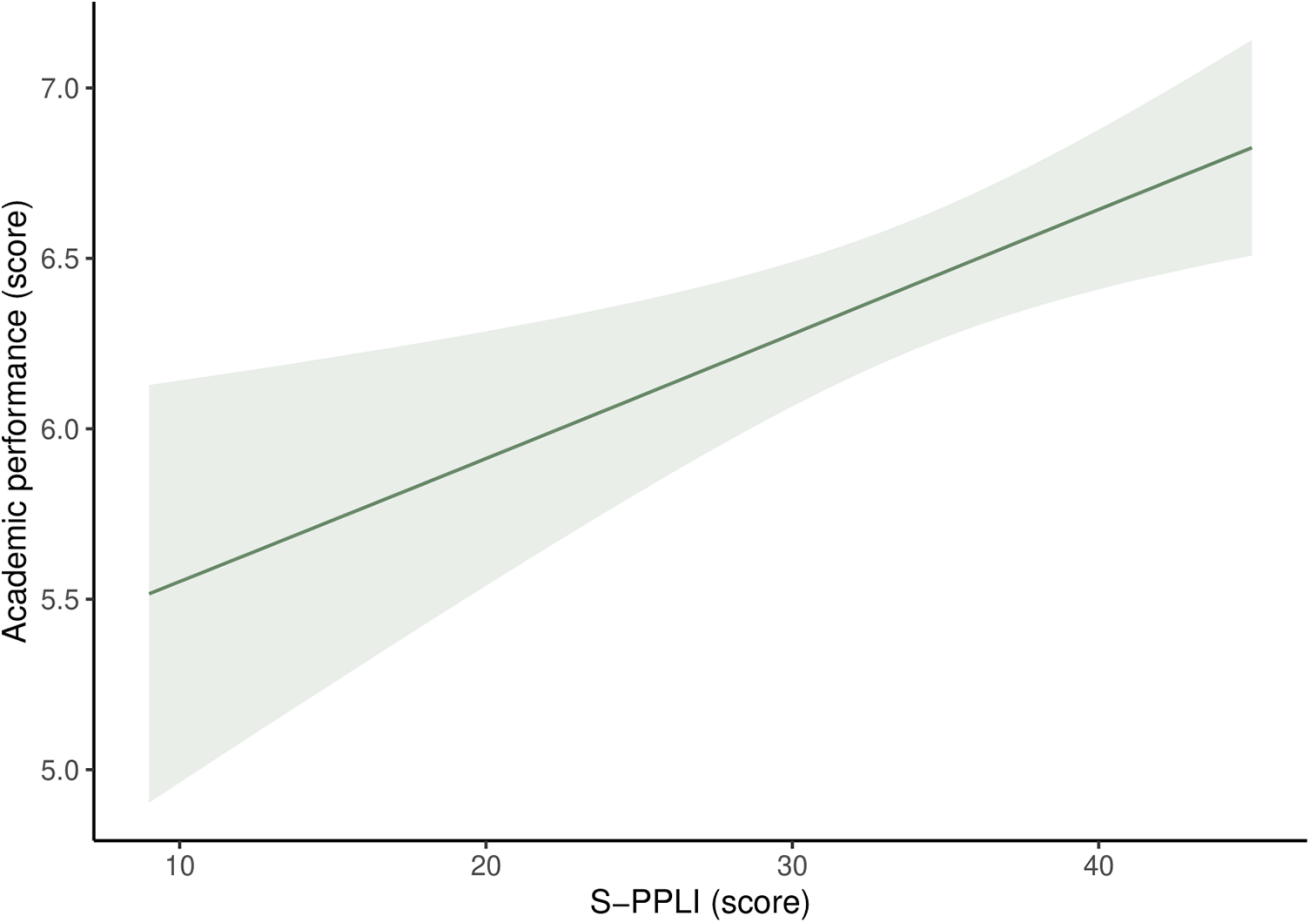
Estimated marginal means of academic performance in adolescents according to perceived physical literacy using generalized additive models. S-PPLI, Spanish Perceived Physical Literacy Instrument. Adjusted for sex, age, socioeconomic status, adherence to the Mediterranean diet, energy intake, physical activity, sedentary behavior, overall sleep duration, and body mass index. Academic performance was calculated as the average of all subjects taken by the adolescents.

Figure 1 indicates the estimated marginal means and 95% CIs of academic performance in relation to the S-PPLI score through smoothed functions derived from GAMs. Overall, a positive trend in the association between S-PPLI scores and academic performance was observed. Furthermore, Supplementary Figure S1 illustrates smoothed functions derived from GAMs that examine academic performance in relation to S-PPLI scores. After closely analyzing the figure and the *edf*, we noticed that the *edf* value is close to 1, which indicates that the relationship resembles a linear term (*F* = 1.11; *edf* = 0.92; *p* = 0.001). Moreover, we identified two significant ranges within the S-PPLI scores. First, there was a significant range between 9 and 33 points on the S-PPLI, indicating an inverse association with academic performance. Second, another significant range was observed at scores of 34 points or higher on the S-PPLI, suggesting a positive association with academic performance.

**Figure 2:**
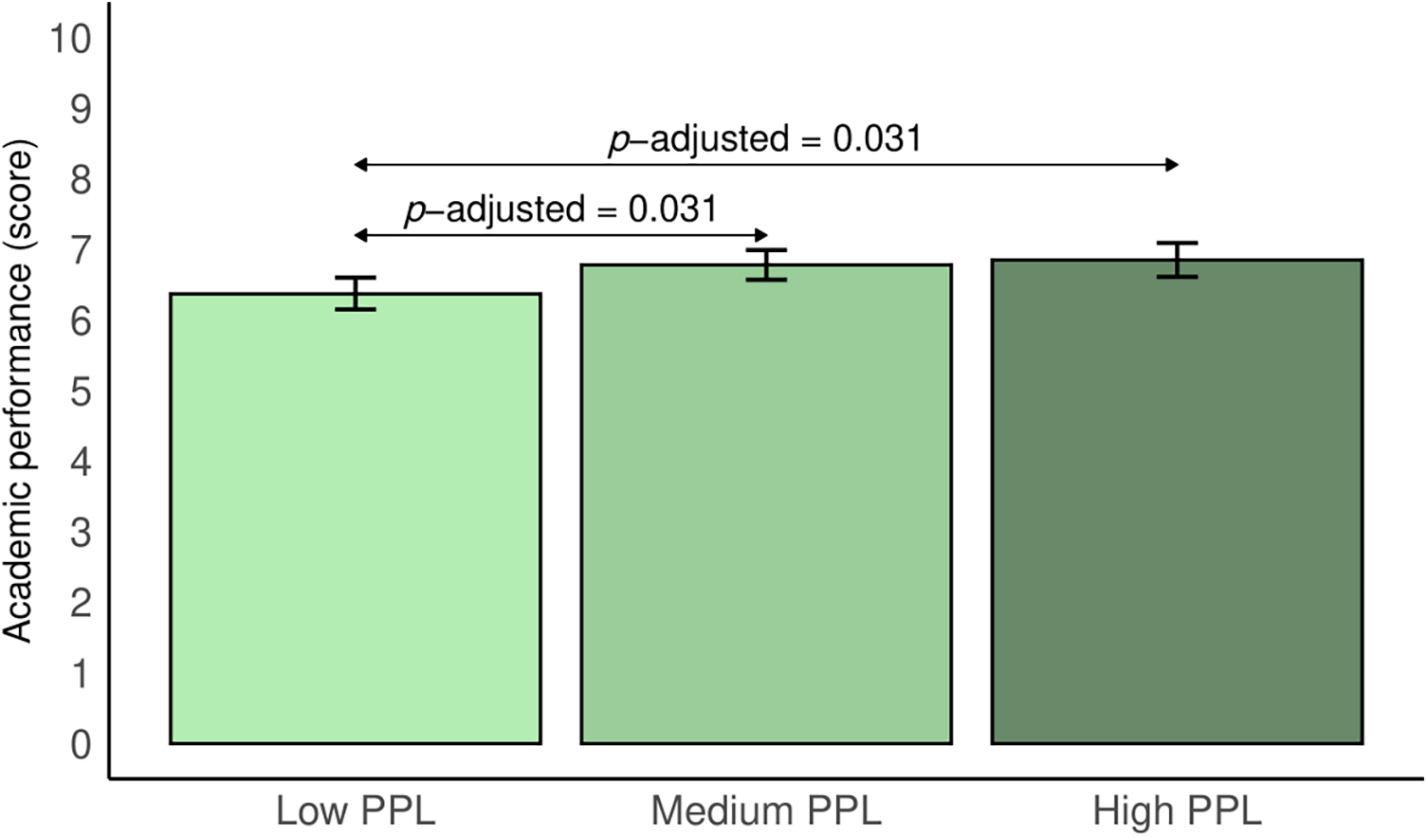
Estimated marginal means of academic performance among adolescents based on their physical literacy status. The data are expressed as estimated marginal means (bars) and bias-corrected and accelerated bootstrapped 95% confidence intervals (lines). Adjusted for sex, age, socioeconomic status, adherence to the Mediterranean diet, energy intake, physical activity, sedentary behavior, overall sleep duration, and body mass index. PPL, perceived physical literacy. Academic performance was calculated as the average of all subjects taken by the adolescents.

Figure 2 indicates the estimated marginal means of academic performance and their BCa bootstrapped 95% CI according to PPL status. Participants with low PPL had the lowest academic performance (mean = 6.4; *BCa* bootstrapped 95% CI: 6.2–6.6). Conversely, those adolescents with high PPL had the highest academic performance (mean = 6.9; *BCa* bootstrapped 95% CI: 6.6–7.1). Significant differences were found between low PPL and medium PPL (*p*-adjusted = 0.031) and between low PPL and high PPL (*p*-adjusted = 0.031). Supplementary Table S1 shows the full estimated marginal means of the analyses of covariance for GPA, language, maths, foreign language (English), and physical education, both for the total sample and stratified by sex. Overall, adolescents with low PPL had the lowest academic scores for GPA, language, mathematics, foreign language (English), and physical activity (both overall and divided by sex). According to sex, significant differences were found between adolescents with low PPL and those with high PPL globally (*p*-adjusted = 0.007) and for boys (*p*-adjusted = 0.033), and a barely detectable significant difference was observed for girls (*p*-adjusted = 0.050) in the physical education grade. In addition, a significant difference was observed when all the samples were analyzed together between adolescents with medium PPL and those with high PPL (*p*-adjusted = 0.014).

## 4 Discussion

Overall, our findings suggest that greater physical literacy is related to greater academic performance in adolescents. Although there is limited evidence on the relationship between physical literacy and academic performance (17–19), these results agree with previous studies found in the literature. For instance, one cross-sectional study revealed a positive association between physical literacy and academic performance among Chinese undergraduates (aged 18–21 years) (19). Another cross-sectional study of United States adolescents suggested that incorporating psychomotor connections with physical literacy could represent a hopeful approach to stimulating changes in physical fitness behavior and establishing a pathway to academic performance for adolescents (17). Similarly, an intervention showed that integrating physical literacy into active school recesses had a positive effect on academic performance (among Chinese children) (18). However, caution is warranted when comparing these findings, due to these authors used different tools to assess physical literacy [e.g., questionnaire (19), tests (17)] and academic performance [e.g., only Chinese and mathematics (18), the State of Texas Assessments of Academic Readiness (17)], different statistical approaches to examine the associations [e.g., linear regression analyses (19), generalized estimating equations (18), one-way multivariate analysis of covariance (17)] and examined children (18) or adult rather than adolescent populations (19). Although only a few studies examining this specific association, there are several potential mechanisms that explain these results.

First, one possible explanation for this finding could be attributed to the concept of greater concentration and attention among individuals with a higher level of physical literacy. When individuals have a solid foundation in physical skills and are comfortable with various physical activities (10), they tend to show greater self-confidence when participating in these activities. This increase in self-confidence and competence can improve students’ cognitive performance, especially in terms of concentration and memory (34). Additionally, research has consistently revealed positive associations between regular engagement in exercise and physical activity and academic performance, cognitive function, brain structure, and brain activity in adolescents (35). This phenomenon may be attributed to the release of endorphins and neurotransmitters through physical exercise, which not only elevates mood but also enhances cognitive function, ultimately contributing to greater academic performance (35).

Second, the link found between physical literacy and academic performance may also be attributed to the development of certain cognitive and personality skills. The practice of physical activities is not only related to physical fitness but also plays a fundamental role in the development of cognitive skills such as planning, decision making, problem solving and motor coordination (36). These skills are often enhanced through regular participation in sports and physical activities and could have a significant impact on a student's academic performance by improving his or her ability to organize tasks, make the right decisions and face academic challenges (37). Moreover, involvement in sports and physical activities can promote essential personality traits such as determination, perseverance, grit, resilience, and critical thinking (38). These qualities are highly transferable to various academic areas and can significantly contribute to a student's success.

Third, the relationship between physical literacy and academic performance can also be explained by the social and discipline-related skills developed through sports and physical activities. Many sports and physical activities emphasize teamwork, communication, and discipline [34]. Adolescents who actively engage in sports often learn how to collaborate effectively as part of a team, follow instructions from coaches, and set and work toward both short-term and long-term goals—skills that prove invaluable in the academic setting (39). This synergy between sports and academics results in a more engaging and enriched learning experience, ultimately contributing to greater academic performance (40). The social, teamwork, and discipline-oriented skills taught through sports can empower students to excel not only in their physical endeavors but also in their pursuit of academic excellence, creating a well-rounded and accomplished educational experience (37).

Fourth, engagement in physical activity can play a crucial role in promoting the establishment of routines among adolescents. Commitment to regular physical activity often necessitates the creation of structured routines and schedules (41). These routines, when consistently followed, can have a positive spillover effect on various aspects of an individual's life, including their approach to academics. Adolescents who engage in physical activity may become more adept at establishing effective study habits and strategies due to the discipline required in maintaining their physical fitness routines (42, 43). Regular schedules for exercise can reinforce the importance of time management and discipline, skills that can be readily transferred to academic tasks. Research conducted by Walck-Shannon et al. (43) has indicated that students who employ more active study strategies and dedicate sufficient time to them tend to perform better on their exams. This suggests that the discipline and time management developed through physical activity can contribute to a more focused and productive approach to studying (44). Additionally, the reorganization of students' daily school schedules to incorporate physical activity and structured routines has been shown to lead to overall improvements in academic performance (42).

Despite the obtained results, it is crucial to interpret this study while keeping certain limitations in mind. Because a cross-sectional approach was used in this study, it is not feasible to establish a direct cause-and-effect relationship based on the findings. Further studies applying different methodologies (e.g., interventions) are needed to elucidate whether greater physical literacy is related to greater academic performance in adolescents. On the other hand, using questionnaires to gather data on PPL and other covariates might introduce bias since differences in willingness to disclose information or inaccuracies in recalling details could impact the results. Conversely, the main strength of this study lies in the assessment of academic performance based on grades provided by secondary schools. It is noteworthy that academic performance, as measured in this study, tends to be more objective and less subject to bias than information reported by adolescents. In addition, this is one of the first studies analyzing the association between physical literacy and academic performance in adolescents. Moreover, our analyses were adjusted for several sociodemographic, lifestyle, and anthropometric variables, enhancing the reliability of our findings.

## 5 Conclusions

Having greater physical literacy could be a relevant factor for achieving greater academic performance in adolescents. Adolescents with low PPL had lower academic performance than did their counterparts with medium or high PPL. This study has potential implications for physical education instructors, school leaders, and healthcare practitioners. Through a skills-based approach, physical education enriched with physical literacy could enhance the opportunities offered to children and adolescents in support of their holistic development and ongoing engagement in physical activity (45), which could serve as a pathway to academic performance for adolescents.

## Data Availability

The raw data supporting the conclusions of this article will be made available by the authors, without undue reservation.

## References

[1] Bücker S Nuraydin S Simonsmeier BA Schneider M Luhmann M. Subjective well-being and academic achievement: a meta-analysis. J Res Pers. (2018) 74:83–94. 10.1016/j.jrp.2018.02.007

[2] Peng P Kievit RA. The development of academic achievement and cognitive abilities: a bidirectional perspective. Child Dev Perspect. (2020) 14(1):15–20. 10.1111/cdep.12352 35909387 PMC7613190

[3] Pizzolato JE Brown EL Kanny MA. Purpose plus: supporting youth purpose, control, and academic achievement. New Dir Youth Dev. (2011) 2011(132):75–88. 10.1002/yd.429 22275280

[4] Zheng LR Atherton OE Trzesniewski K Robins RW. Are self-esteem and academic achievement reciprocally related? Findings from a longitudinal study of Mexican-origin youth. J Pers. (2020) 88(6):1058–74. 10.1111/jopy.12550 32368788

[5] Jiang MM Gao K Wu ZY Guo PP. The influence of academic pressure on adolescents’ problem behavior: chain mediating effects of self-control, parent–child conflict, and subjective well-being. Front Psychol. (2022) 13:954330. 10.3389/fpsyg.2022.954330 36211862 PMC9534181

[6] Busch V Loyen A Lodder M Schrijvers AJP van Yperen TA de Leeuw JRJ. The effects of adolescent health-related behavior on academic performance: a systematic review of the longitudinal evidence. Rev Educ Res. (2014) 84(2):245–74. 10.3102/0034654313518441

[7] Burrows T Goldman S Pursey K Lim R. Is there an association between dietary intake and academic achievement: a systematic review. J Hum Nutr Diet. (2017) 30(2):117–40. 10.1111/jhn.12407 27599886

[8] Haverkamp BF Wiersma R Vertessen K Van Ewijk H Oosterlaan J Hartman E. Effects of physical activity interventions on cognitive outcomes and academic performance in adolescents and young adults: a meta-analysis. J Sports Sci. (2020) 38(23):2637–60. 10.1080/02640414.2020.1794763 32783695

[9] Longmuir PE Tremblay MS. Top 10 research questions related to physical literacy. Res Q Exerc Sport. (2016) 87(1):28–35. 10.1080/02701367.2016.1124671 26889582

[10] Tremblay MS Costas-Bradstreet C Barnes JD Bartlett B Dampier D Lalonde C Canada’s physical literacy consensus statement: process and outcome. BMC Public Health. (2018) 18(S2):1034. 10.1186/s12889-018-5903-x 30285701 PMC6167775

[11] Edwards LC Bryant AS Keegan RJ Morgan K Definitions JA. Foundations and associations of physical literacy: a systematic review. Sports Med. (2017) 47(1):113–26. 10.1007/s40279-016-0560-7 27365029 PMC5215133

[12] Whitehead M, editor. Physical Literacy Across the World. 1st ed. London: Routledge (2019). Disponible en: https://www.taylorfrancis.com/books/9781351334136 (citado de febrero de 23, 2023)

[13] Whitehead M. Physical Literacy: Throughout the Lifecourse. 1st ed. London, United Kingdom; New York, United States: Routledge (2010). (International studies in physical education and youth sport).

[14] Chen A. Operationalizing physical literacy for learners: embodying the motivation to move. J Sport Health Sci. (2015) 4(2):125–31. 10.1016/j.jshs.2015.03.005

[15] Giblin S Collins D Button C. Physical literacy: importance, assessment and future directions. Sports Med. (2014) 44(9):1177–84. 10.1007/s40279-014-0205-7 24898813

[16] Grauduszus M Wessely S Klaudius M Joisten C. Definitions and assessments of physical literacy among children and youth: a scoping review. BMC Public Health. (2023) 23(1):1746. 10.1186/s12889-023-16680-x 37679785 PMC10486121

[17] Gu X Zhang T Lun (Alan) Chu T Zhang X Thomas Thomas K. Do physically literate adolescents have better academic performance? Percept Mot Skills. (2019) 126(4):585–602. 10.1177/0031512519845274 31033404

[18] Zhang D Shi L Zhu X Chen S Liu Y. Effects of intervention integrating physical literacy into active school recesses on physical fitness and academic achievement in Chinese children. J Exerc Sci Fit. (2023) 21(4):376–84. 10.1016/j.jesf.2023.09.004 PMC10624586 37927355

[19] Ma RS Sum RKW Li MH Huang Y Niu XL. Association between physical literacy and physical activity: a multilevel analysis study among Chinese undergraduates. IJERPH. (2020) 17(21):7874. 10.3390/ijerph17217874 33121068 PMC7663683

[20] Lundvall S. Physical literacy in the field of physical education—a challenge and a possibility. J Sport Health Sci. (2015) 4(2):113–8. 10.1016/j.jshs.2015.02.001

[21] Cairney J Dudley D Kwan M Bulten R Kriellaars D. Physical literacy, physical activity and health: toward an evidence-informed conceptual model. Sports Med. (2019) 49(3):371–83. 10.1007/s40279-019-01063-3 30747375

[22] García-Hermoso A Ramírez-Vélez R Lubans DR Izquierdo M. Effects of physical education interventions on cognition and academic performance outcomes in children and adolescents: a systematic review and meta-analysis. Br J Sports Med. (2021) 55(21):1224–32. 10.1136/bjsports-2021-104112 34187782

[23] Rico-González M. The effect of primary school-based physical education programs: a systematic review of randomized controlled trials. J Phys Act Health. (2023) 20(4):317–47. 10.1123/jpah.2022-0452 36870346

[24] López-Gil JF. The eating healthy and daily life activities (EHDLA) study. Children. (2022) 9(3):370. 10.3390/children9030370 35327742 PMC8947596

[25] López-Gil JF Martínez-Vizcaíno V Tárraga-López PJ García-Hermoso A. Cross-cultural adaptation, reliability, and validation of the spanish perceived physical literacy instrument for adolescents (S-PPLI). J Exerc Sci Fitness. (2023) 21(3):246–52. 10.1016/j.jesf.2023.03.002 PMC10182311 37193580

[26] Sum RKW Ha ASC Cheng CF Chung PK Yiu KTC Kuo CC Construction and validation of a perceived physical literacy instrument for physical education teachers. PLoS One. (2016) 11(5):e0155610. 10.1371/journal.pone.0155610 27195664 PMC4873233

[27] Currie C Molcho M Boyce W Holstein B Torsheim T Richter M. Researching health inequalities in adolescents: the development of the health behaviour in school-aged children (HBSC) family affluence scale. Soc Sci Med. (2008) 66(6):1429–36. 10.1016/j.socscimed.2007.11.024 18179852

[28] Serra-Majem L Ribas L Ngo J Ortega RM García A Pérez-Rodrigo C Food, youth and the Mediterranean diet in Spain. Development of KIDMED, Mediterranean diet quality Index in children and adolescents. Public Health Nutr. (2004) 7(7):931–5. 10.1079/PHN2004556 15482620

[29] Rodríguez IT Ballart JF Pastor GC Jordà EB Val VA. Validation of a short questionnaire on frequency of dietary intake: reproducibility and validity. Nutr Hosp. (2008) 23(3):242–52. 18560701

[30] Saint-Maurice PF Welk GJ. Validity and calibration of the youth activity profile. PLoS One. (2015) 10(12):e0143949. 10.1371/journal.pone.0143949 26630346 PMC4668067

[31] Wood SN. Fast stable restricted maximum likelihood and marginal likelihood estimation of semiparametric generalized linear models. J R Stat Soc B Stat Methodol. (2011) 73(1):3–36. 10.1111/j.1467-9868.2010.00749.x

[32] Marra G Wood SN. Practical variable selection for generalized additive models. Comput Stat Data Anal. (2011) 55(7):2372–87. 10.1016/j.csda.2011.02.004

[33] Benjamini Y Hochberg Y. Controlling the false discovery rate: a practical and powerful approach to multiple testing. J R Stat Soc Ser B Methodol. (1995) 57(1):289–300. 10.1111/j.2517-6161.1995.tb02031.x

[34] Centers for Disease Control and Prevention. Comprehensive School Physical Activity Programs: a Guide for Schools. Atalanta, United States: Department of Health and Human Services (2013). Disponible en: https://www.cdc.gov/healthyschools/physicalactivity/pdf/13_242620-A_CSPAP_SchoolPhysActivityPrograms_Final_508_12192013.pdf (Accessed November 01, 2023).

[35] Herting MM Chu X. Exercise, cognition, and the adolescent brain. Birth Defects Research. (2017) 109(20):1672–9. 10.1002/bdr2.1178 29251839 PMC5973814

[36] Mandolesi L Polverino A Montuori S Foti F Ferraioli G Sorrentino P Effects of physical exercise on cognitive functioning and wellbeing: biological and psychological benefits. Front Psychol. (2018) 9:509. 10.3389/fpsyg.2018.00509 29755380 PMC5934999

[37] Pinto-Escalona T Valenzuela PL Esteban-Cornejo I Martínez-de-Quel Ó. Sport participation and academic performance in young elite athletes. IJERPH. (2022) 19(23):15651. 10.3390/ijerph192315651 36497726 PMC9737165

[38] Whitley MA Massey WV Wilkison M. A systems theory of development through sport for traumatized and disadvantaged youth. Psychol Sport Exerc. (2018) 38:116–25. 10.1016/j.psychsport.2018.06.004

[39] Lee O Park M Jang K Park Y. Life lessons after classes: investigating the influence of an afterschool sport program on adolescents’ life skills development. Int J Qual Stud Health Well-Being. (2017) 12(1):1307060. 10.1080/17482631.2017.1307060 28367697 PMC5421366

[40] Muñoz Miguel JP Simón De Blas C Anguita Rodríguez F García Sipols AE. Collaborative learning in management subjects to university students: a multi-level research to identify group profile, engagement and academic performance. Int J Manag Educ. (2023) 21(1):100762. 10.1016/j.ijme.2022.100762

[41] Arlinghaus KR Johnston CA. The importance of creating habits and routine. Am J Lifestyle Med. (2019) 13(2):142–4. 10.1177/1559827618818044 30800018 PMC6378489

[42] Williams KM Shapiro TM. Academic achievement across the day: evidence from randomized class schedules. Econ Educ Rev. (2018) 67:158–70. 10.1016/j.econedurev.2018.10.007

[43] Walck-Shannon EM Rowell SF Frey RF. To what extent do study habits relate to performance? Knight J, editor. LSE. (2021) 20(1):ar6. 10.1187/cbe.20-05-0091 PMC8108503 33444109

[44] Kohl HW Cook HD. Physical activity, fitness, and physical education: effects on academic performance. In: Kohl HW III Cook HD, editors. Educating the Student Body: Taking Physical Activity and Physical Education to School [Internet]. Washington, DC: National Academies Press (2013). p. 161–196. Available from: http://www.nap.edu/catalog/18314 (Accessed January 14, 2024). 24851299

[45] Durden-Myers E Bartle G. Physical-literacy-enriched physical education: a capabilities perspective. Children. (2023) 10(9):1503. 10.3390/children10091503 37761464 PMC10527893

