# Supplementary material for: Exploring the link between perceived physical literacy and academic performance outcomes: insights from the EHDLA study
Source: Front Sports Act Living. 2024 Jan 24;6:1352114. doi: 10.3389/fspor.2024.1352114 (PMC10851748; doi:10.3389/fspor.2024.1352114)
Supplement: Supplementary file 1 [file Table1.docx]

**Table S1.** Estimated marginal means of academic performance among adolescents based on their physical literacy status.

|  | **Total sample** | | | **Boys** | | | **Girls** | | |
| --- | --- | --- | --- | --- | --- | --- | --- | --- | --- |
| **Subject** | **Low PPL**  (9-31 points)  (*n* = 266; 33.9%) | **Medium PPL**  (32-36 points)  (*n* = 285; 36.3%) | **High PPL**  (37-45 points)  (*n* = 234; 29.8%) | **Low PPL**  (12-32 points)  (*n* = 126; 36.0%) | **Medium PPL**  (33-36 points)  (*n* = 106; 30.3%) | **High PPL**  (37-45 points)  (*n* = 118; 33.7%) | **Low PPL**  (9-30 points)  (*n* = 134; 30.8%) | **Medium PPL**  (31-35 points)  (*n* = 155; 35.6%) | **High PPL**  (36-45 points)  (*n* = 146; 33.6%) |
| GPA (score) | 6.4 (6.2-6.6) | 6.8 (6.6-7.0) ^a^ | 6.9 (6.6-7.1) ^a^ | 6.2 (5.9-6.5) | 6.5 (6.2-6.8) | 6.7 (6.3-7.1) | 6.6 (6.3-7.0) | 7.0 (6.7-7.2) | 7.0 (6.8-7.3) |
| Language (score) | 6.2 (5.9-6.5) | 6.5 (6.2-6.8) | 6.6 (6.2-6.8) | 5.9 (5.5-6.3) | 6.0 (5.6-6.5) | 6.3 (5.8-6.7) | 6.6 (6.1-7.0) | 6.8 (6.5-7.2) | 6.8 (6.5-7.2) |
| Maths (score) | 5.5 (5.2-5.8) | 6.0 (5.7-6.2) | 5.9 (5.5-6.2) | 5.5 (5.1-5.9) | 5.9 (5.4-6.3) | 5.6 (5.1-6.2) | 5.6 (5.1-6.0) | 6.0 (5.6-6.4) | 6.0 (5.6-6.4) |
| Foreign language (score) ^†^ | 6.1 (5.7-6.4) | 6.4 (6.1-6.6) | 6.2 (6.0-6.5) | 5.9 (5.5-6.3) | 6.0 (5.5-6.4) | 6.1 (5.7-6.5) | 6.3 (5.9-6.7) | 6.6 (6.3-7.0) | 6.4 (6.0-6.7) |
| Physical education (score) | 6.7 (6.4-6.9) | 7.1 (6.8-7.3) ^a^ | 7.5 (7.2-7.8) ^a,b^ | 6.6 (6.2-7.0) | 6.9 (6.5-7.2) | 7.6 (7.2-7.9) ^a^ | 6.8 (6.4-7.2) | 7.1 (6.8-7.4) | 7.4 (7.1-7.7) |

The data are expressed as estimated marginal means (bars) and bias-corrected and accelerated bootstrapped 95% confidence intervals (lines). Adjusted for sex, age, socioeconomic status, adherence to the Mediterranean diet, energy intake, physical activity, sedentary behavior, overall sleep duration, and body mass index. GPA, grade point average; PPL, perceived physical literacy. The GPA was calculated as the average of all the measurements taken by the adolescents. ^†^ English as a foreign language. ^a^ Statistically significant difference compared to adolescents with low PPL (*p* < 0.05). ^b^ Statistically significant difference compared to adolescents with medium PPL (*p* < 0.05).
