# Supplementary material for: Exploring the link between perceived physical literacy and academic performance outcomes: insights from the EHDLA study
Source: Front Sports Act Living. 2024 Jan 24;6:1352114. doi: 10.3389/fspor.2024.1352114 (PMC10851748; doi:10.3389/fspor.2024.1352114)
Supplement: Supplementary file 2 [file Datasheet1.docx]

**Figure S1.** Relationship between perceived physical literacy and academic performance in adolescents using generalized additive models. GPA, grade point average; S-PPLI, Spanish Perceived Physical Literacy Instrument. Adjusted for sex, age, socioeconomic status, adherence to the Mediterranean diet, energy intake, physical activity, sedentary behavior, overall sleep duration, and body mass index. The GPA was calculated as the average of all the measurements taken by the adolescents.
